# Cardioprotective and Antihypertensive Effects of Topical Capsaicin in a Rat Model

**DOI:** 10.3390/antiox14080966

**Published:** 2025-08-06

**Authors:** Juan Carlos Torres-Narváez, Vicente Castrejón-Téllez, María Sánchez-Aguilar, Agustina Cano-Martínez, Elizabeth Soria-Castro, Julieta Anabell Díaz-Juárez, Israel Pérez-Torres, Verónica Guarner-Lans, Elvira Varela-López, María de la Luz Ibarra-Lara, Gabriela Zarco-Olvera, Alvaro Vargas-González, Pedro L. Flores-Chávez, Leonardo del Valle-Mondragón

**Affiliations:** 1 Departamento de Farmacología Dr. Rafael Méndez Martínez, Instituto Nacional de Cardiología Ignacio Chávez, Juan Badiano No. 1, Col. Sección XVI, Tlalpan, Ciudad de México 14080, Mexico; juancarlostn63@hotmail.com (J.C.T.-N.); msanchezaguilar@gmail.com (M.S.-A.); anabelldij@gmail.com (J.A.D.-J.); luzibarralara@gmail.com (M.d.l.L.I.-L.); gabriela0304@hotmail.com (G.Z.-O.); 2 Departamento de Fisiología, Instituto Nacional de Cardiología Ignacio Chávez, Juan Badiano No. 1, Col. Sección XVI, Tlalpan, Ciudad de México 14080, Mexico; vcastrejn@yahoo.com.mx (V.C.-T.); cmamx2002@yahoo.com.mx (A.C.-M.); gualanv@yahoo.com (V.G.-L.); alvaro.vargas@cardiologia.org.mx (A.V.-G.); 3 Departamento de Biomedicina Cardiovascular, Instituto Nacional de Cardiología Ignacio Chávez, Juan Badiano No. 1, Col. Sección XVI, Tlalpan, Ciudad de México 14080, Mexico; elizabeth.soria@cardiologia.org.mx (E.S.-C.); pertorisr@yahoo.com.mx (I.P.-T.); 4 Laboratorio de Cardiología Translacional, Unidad de Investigación en Medicina Translacional UNAM/INCAR, Instituto Nacional de Cardiología Ignacio Chávez, Juan Badiano No. 1, Col. Sección XVI, Tlalpan, Ciudad de México 14080, Mexico; varelopz@yahoo.com; 5 Departamento de Instrumentación Electromecánica, Instituto Nacional de Cardiología Ignacio Chávez, Juan Badiano No. 1, Col. Sección XVI, Tlalpan, Ciudad de México 14080, Mexico; pelfoch07@yahoo.com.mx

**Keywords:** hypertension, capsaicin, TRPV1, CGRP, cell damage, inflammation

## Abstract

TRPV1 regulates neuronal and vascular function mediated by NO and CGRP. Systemic arterial hypertension (SAH) induces an imbalance in vascular mediators NO and CGRP by altering the transport of Ca^2+^ ions through TRPV1, generating cellular damage. We studied the effect of topical capsaicin (CS) treatment on cardiac mechanical work, oxidative stress (TAC, NO, BH4, and BH2), cellular damage (MDA, MTO, and 8HO2dG), and inflammation (IL-6 and TNFα), generated by SAH, which was induced by *L*-NAME, in male Wistar rats. CS was added to a moisturizing cream and applied to the abdomen of animals for two weeks. Experimental groups were as follows: (1) Control, (2) Control+Cream, (3) Hypertensive, and (4) Hypertensive+Cream. Hearts were exposed to ischemia-reperfusion (I-R) using the Langendorff technique to study the potential cardioprotection of CS. Expression of SOD1, SOD2, catalase, eNOS, pNOS, TRPV1, and CGRP in cardiac tissue was evaluated. In the Hypertensive group, TRPV1 activation by CS (Hypertensive+Cream) reduced oxidative stress (OS), decreasing cellular damage and inflammation and increasing TAC, modulating biochemical and tissue alterations induced by OS generated by SAH. In parallel, an increase in tissue levels and the expression of CGRP, TRPV1, and eNOS, induced by CS, was observed. These findings indicate that pretreatment with CS attenuates cardiac I-R and SAH injury in rats. The cardioprotective mechanism may be based on TRPV1-mediated CGRP overexpression.

## 1. Introduction

Systemic arterial hypertension (SAH) is one of the greatest public health challenges worldwide. In recent decades, lifestyle changes have led to stress, sedentary lifestyles, and poor eating habits, contributing to an increase in cardiovascular comorbidities such as obesity, diabetes mellitus, kidney damage, and vascular disease [1].

Despite its high prevalence, hypertension remains underdiagnosed, and this condition remains poorly controlled. It is estimated that nearly 50% of hypertensive patients are unaware of their condition, and among those who receive treatment, only a fraction manage to maintain their blood pressure levels within appropriate ranges [2].

In SAH, high blood pressure levels are associated with an increase in reactive oxygen species (ROS), which generates oxidative stress (OS) and consequently an alteration of vascular homeostasis, resulting in endothelial dysfunction and decreased bioavailability of nitric oxide (NO) [2,3,4].

Increased ROS produces an imbalance in the endogenous antioxidant system, causing damage to membrane lipids, proteins, and other macromolecules in cells and tissues [5,6]. In extreme cases, it inhibits mitochondrial respiration, thereby interrupting the Krebs cycle, generating intracellular hypoxia and a decrease in ATP production (mitochondrial dysfunction). In addition, it causes damage to nuclear DNA [7,8]. This leads to apoptosis and, ultimately, acute myocardial infarction (AMI) [9,10].

On the other hand, transient receptor potential vanilloid type 1 (TRPV1) is a non-selective cation channel that allows for the passage of Ca^2+^, Na^+^, and K^+^ through membranes of various cell types, such as neurons, endothelium, and vascular smooth muscle, and of organelles, such as rough endoplasmic reticulum and mitochondria. TRPV1 is activated through endogenous stimuli such as changes in pH, temperature, and shear stress, as well as by molecules such as anandamide and 17*β*-estradiol. TRPV1 can be exogenously activated or inactivated by pharmacological agents, and capsaicin (CS) constitutes an activator of the receptor at low doses but an inhibitor at high doses [11]. In turn, prolonged stimulation by its agonist (CS) causes the receptor to enter a refractory period in which it becomes unable to respond to any stimulus, finding itself in a desensitized state. Depending on the type of agonist, dose, and exposure time, this period can last from minutes to days. Prolonged exposure to vanilloid agonists induces endocytosis of the TRPV1 receptor in a dose- and time-dependent manner. Capsaicin-induced TRPV1 internalization has been shown to require channel activation and Ca^+2^ influx through pores [11,12].

**Figure 1:**
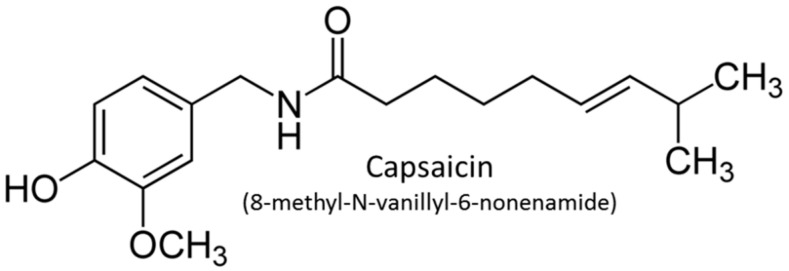
Molecular structure of capsaicin (CS). Chem Sketch 2024.1.0. Advanced Chemistry Development, Inc. (ACD/Labs).

CS is an organic polyphenol-type compound (Figure 1) that gives chili peppers their spicy flavor. It is used to study functional and therapeutic properties of TRPV1 in diseases related to pain, cardiovascular disease [12,13,14,15], hypertension, and breast cancer, among others [11,16,17,18].

Regulatory actions of OS and vasodilation through TRPV1 activation are mediated by poorly understood mechanisms. They involve action of neuropeptides such as substance P and calcitonin gene-related peptide (CGRP), which, along with NO, have important vasodilatory properties, particularly in the heart [19,20].

We have recently reported that subcutaneous administration of CS for 4 days has a preventive effect against damage due to NO-deficiency in SAH, and it also protects against ischemia-reperfusion (I-R) damage in rats. This effect occurs by restoring the NO pathway. Subcutaneous CS treatment resulted in gradual drug absorption, but it caused mild injury at the application site [21,22]. Currently, we are interested in delivering CS to the bloodstream via topical administration of a CS-impregnated moisturizer [23,24], which is a less invasive strategy.

Experimentally, TRPV1 activation occurs at doses of less than 1% of CS. Furthermore, at this concentration, there is a decrease in animal body weight, as well as a reduction in blood pressure and cardioprotection [25,26].

The objective of this study was to evaluate whether TRPV1 activation by topical treatment with CS can generate cardioprotection in SAH and I-R, through regulation of systemic levels of metabolites related to OS, cellular damage, and inflammation [2,3,25], as well as through CGRP expression.

Our proposed hypothesis is based on restoration by CS of the unbalanced flow of Ca^2+^ ions across the cell membrane (an alteration induced by SAH) and by modulation of TRPV1 when this agonist circulates systemically. This diminishes OS, thus increasing the bioavailability of NO and, therefore, CGRP. This results in reduced lipid peroxidation of cell membranes and tissue inflammation.

## 2. Materials and Methods

### 2.1. Reagents

CS (8-methyl-*N*-vanillyl-6-nonenamide), *L*-nitro-arginine methyl ester (*L*-NAME, NO synthase inhibitor), and all reagents used in this study were obtained from Sigma-Aldrich Chemical Co., Ltd., St. Louis, MO, USA.

The moisturizing base used to prepare topical CS cream (30 µM, CS 120 mg/400 g cream) was obtained from Hydro Meritor, S.A. de C.V., Mexico City, Mexico.

### 2.2. Preparation of Cream with Capsaicin

To prepare the CS cream, 120 mg of CS was diluted in 3 mL of 70% ethanol (21) and then added to a semi-solid mixer (Power 750w, Oster México, S.A. de C.V., Querétaro, Mexico) containing 400 g of moisturizing base cream. The mixture was mixed at 10 rpm for 3 h, monitoring CS concentration every 15 min. When the CS concentration was homogeneous, cream was poured into airtight Teflon containers and stored away from light and under normal temperature conditions (25 ± 1.5 °C).

### 2.3. Animals

Experimental animals (male Wistar rats weighing 300 to 350 g) were obtained from the Bioterium Department of Ignacio Chávez National Institute of Cardiology in Mexico City, after approval by the Institutional Committee for Care of Laboratory Animals (CICUAL) and the Institutional Ethics Committee (CIE) on the use and care of experimental animals.

Animals were maintained under standard light conditions (12 h light/dark), at controlled temperature (25 ± 3 °C) and humidity (50 ± 10%). They had free access to a standard diet (certified rat diet, LabDiet 5001, PMI Nutrition International, Richmond, IN, USA) and water. All procedures were carried out in accordance with guidelines established in Federal Standard for Animal Experimentation and Care (SAGARPA, NOM-062-ZOO-1999, Mexico City, Mexico).

Topical administration was performed on the rat’s abdomen (previously shaved, approximately 5 cm^2^) twice daily for two weeks (2 g of cream = 0.6 mg of CS per daily application).

### 2.4. Experimental Groups

Animals were randomly divided to form 4 groups of 8 animals each: (1) Control, (2) Control+Cream, (3) Hypertensive, and (4) Hypertensive+Cream.

### 2.5. Systemic Arterial Hypertension Induction

SAH was preinduced in rats in groups 3 and 4 with *L*-NAME (200 mg/L) in drinking water for 40 days. From day 26 of starting *L*-NAME treatment until day 40 (last day of uninterrupted *L*-NAME treatment), cream was applied daily topically to the abdominal area. Animals were sacrificed on day 40.

To verify that the *L*-NAME treatment induced SAH, mean arterial pressure was measured in each group using a pneumatic pressure monitor attached to the base of the animals’ tail (Surgi Vet V60046 NIBP, Smiths Medical PM, Inc., Veterinary, Waukesha, WI, USA). This was performed at the beginning and end of treatment. It should be noted that the equipment used only provides mean arterial pressure data.

### 2.6. Determination of Capsaicin

In a 1 mL Eppendorf tube, 30 μL of rat serum, 30 μL of phosphate buffer (pH 7.0), and 30 μL of methyl isobutyl ketone were placed. The mixture was gently homogenized, and a tube was placed under orbital shaking at 10 rpm for 10 min (IKA MS3 Digital, IKA, Co., Ltd., Urbana, IL, USA). It was then centrifuged at 2000 rpm for 10 min (Spectra Fuge 24D LabNet, Co., Ltd., Pasadena, CA, USA) at room temperature. Supernatant was transferred to a 1 mL Eppendorf tube, and an uncapped tube was placed in a 40 °C dry bath (MRC, LabGenius Co., Ltd., Drummond, London, UK) and left there until completely dry. Once the tube was dry, it was capped and stored at −70 °C until analysis.

On the day of analysis, dried residue was reconstituted with 200 µL of HPLC-grade acetonitrile and placed under orbital shaking (IKA MS3 Digital, IKA, Co., Ltd., Urbana, IL, USA) at 20 rpm for 30 min. It was filtered through a Millex-GV filter with a hydrophilic Durapore PVDF membrane with a pore size of 0.22 microns and a diameter of 4 mm (Millipore, Billerica, MA, USA) and subsequently diluted 1:1 with 0.1 M sodium hydroxide.

The resulting mixture was filtered through a 0.22 µm nitrocellulose membrane filter (Corning Inc., Corning, NY, USA) and directly analyzed by UPLC using an ACQUITY UPLC H Class Plus system (Waters Corporation, Milford, MA, USA), coupled to a UV-Visible diode array detector and equipped with an Acquity UPLC BEH300 C18 column (1.7 µm, 21 × 50 mm/Waters Co., Ltd., Milford, MA, USA). Separation was carried out by passing a mixture of 40% A and 60% B, where A corresponded to a 0.02% trifluoroacetic acid solution in water and B to a 0.018% trifluoroacetic acid solution in acetonitrile, at a flow rate of 0.2 mL/min, with detection at 214 nm at 40 °C [21].

### 2.7. Treatment

The Control group and the Hypertensive group of rats were treated topically in the presence and absence of CS cream.

To verify that the topical administration of CS (2 g cream, CS = 0.6 g) achieved a plasma concentration of 22 to 35% of administered dose (approximately 0.132 to 0.210 g CS), as reported in the literature, a group of control rats (n = 3) was randomly selected and subjected to a 3 h (180 min) adsorption time curve following administration of CS cream. To this end, blood samples were taken from rats by cardiac puncture after the animals were anesthetized with sodium pentobarbital (60 mg/kg of body weight). Sampling times were at time zero (without administration of cream) and every 30 min until 3 h (180 min) of adsorption time was completed. It should be noted that, for each point on the curve, 3 animals were sacrificed.

### 2.8. Samples

Rats were anesthetized with sodium pentobarbital (60 mg/kg body weight). Blood samples were collected and centrifuged at 3000 rpm for 5 min at 4 °C. Sera were stored at −70 °C until analysis.

Prior to analysis, sera were deproteinized with cold methanol (analytical grade) and centrifuged at 16,000× *g* (Spectra Fuge 24D, Lab Net International Inc., Edison, NJ, USA) for 15 min at 4 °C. Supernatant was stored at −70 °C for further analysis (samples were used to evaluate biomarkers indicating NO viability (EO), cellular damage, and systemic inflammation).

Tissue sections were also taken from the left ventricle of rat hearts (hearts used were obtained from anesthetized and sacrificed rats). These were washed in cold isotonic saline, sectioned into small pieces, frozen in liquid nitrogen, and stored at −70 °C until the day of analysis (samples were used to assess the same biomarkers in rats heart tissue).

Prior to analysis, 40–50 mg of frozen tissue samples was homogenized in 1000 µL of cold 100 mM phosphate buffer at pH 7.4. Tissue was then centrifuged at 16,000× *g* (Spectra Fuge 24D, Lab Net International Inc., Edison, NJ, USA) for 15 min at 4 °C. Supernatant was removed from the pellet and stored at −70 °C for further analysis (samples were used to assess same biomarkers as in rat serum).

### 2.9. Measurement of Mechanical Activity of the Heart

The mechanical activity of the heart and the role of TRPV1 in preventing damage caused by SAH and I-R were evaluated using the Langendorff isolated heart model. Briefly, the heart was removed and connected to the perfusion system through the ascending aorta. Physiological Krebs solution (120 mM NaCl, 23.4 mM NaHCO_3_, 4.8 mM KCl, 1.2 mM KH_2_PO_4_, 0.86 mM MgSO_4_, 1.25 mM CaCl_2_, and 6.1 mM glucose at pH 7.4, 37 °C, and CO_2_/O_2_ 5:95%. Gas mixture was continuously bubbled into the Krebs solution tank) was retrogradely perfused into the coronary arteries using a peristaltic pump (SAD22, Grass Instruments Co., Ltd., Quincy, MA, USA) at a perfusion rate of 13 mL/min. Left ventricular pressure (LVP) and heart rate (HR) were recorded to calculate cardiac mechanical work (CMW) using the following equation: CMW = LVP × HR. After 30 min of cardiac adaptation and 30 min of control perfusion, a 30 min period of global ischemia (by turning off the perfusion pump) and a 60 min period of reperfusion (by turning the perfusion pump on) were applied. An additional group of normotensive animals, in which isolated hearts did not undergo a period of I-R, was used to demonstrate that cardiac work did not undergo significant modifications during a two-hour experiment [22,27].

Left ventricular pressure was obtained by measuring the mechanical impulse of the heart (contraction), transformed into an electrical impulse, using a latex balloon (internal balloon pressure was 5–10 mmHg) inserted into the left ventricle through the mitral valve and connected to a hydropneumatic transducer (Grass Instruments Co., Ltd., Quincy, MA, USA). Systolic and diastolic pressure data were recorded using a computer system (Grass Poly View, Grass Instruments Co., Ltd., Quincy, MA, USA). From these recordings, heart rate (HR) and cardiac mechanical work (CMW) were obtained, as indicated in the previous paragraph.

Perfusion pressure (PP) was also obtained using a hydropneumatic pressure transducer (Grass Instruments Co., Ltd., Quincy, MA, USA) connected directly to the perfusion system. From these recordings, coronary vascular resistance (CVR) was obtained using the following equation: CVR = PP/Perfusion rate.

### 2.10. Biomarker Measurement

Evaluation was performed on serum and ventricular tissue from different experimental groups. On the day of analysis, samples (serum and tissue homogenate supernatants) were gradually thawed and processed according to each analysis protocol.

Methodologies used for biomarker quantification were applied identically to all sample types.

Thus, NO bioavailability in HAS-induced OS was evaluated by quantifying biomarkers TAC, NO, BH4, BH2, CGRP, and OxCap (oxidant capacity) under control, hypertensive, treated control, and treated hypertensive conditions.

In addition, damage to cell membranes and nuclear DNA was assessed by quantifying biomarkers MDA, MTO, and 8HO2dG. The main inflammatory factors (IL-6 and TNF-α) were also evaluated.

In parallel, the expression of endogenous antioxidant enzymes SOD1, SOD2, and catalase was determined to corroborate a possible alteration in their expression due to the effect of HAS induced by *L*-NAME and, if applicable, whether CS could regulate them.

Expression of eNOS and pNOS was also evaluated to verify whether NO synthesis was affected by SAH and whether CS could correct the alteration.

TRPV1 and CGRP were assessed by immunohistochemistry in cardiac tissue from different study groups. Areas of heart sections examined were epicardial, myocardial, endocardial, and transverse septal fibers. This was completed to verify whether CS could increase its expression after its decrease due to *L*-NAME-induced SAH.

### 2.11. Nitric Oxide

To 20 µL of the sample, 100 µL of 0.8% vanadium III chloride in 1 M phosphoric acid was added. The sample was homogenized, and 50 µL of 2% sulfanilamide in 5% phosphoric acid was added. Subsequently, 50 µL of 0.2% N-(1-naphthyl)-ethylenediamine in distilled water was added, and the mixture was gently homogenized. The mixture was left to stand for 50 min, protected from light. After incubation time, samples were analyzed spectrophotometrically at 572 nm and then at 587 nm (UV-1800 spectrophotometer, Shimadzu LA, S.A. Buenos Aires, Argentina). NO concentration was obtained by preparing a moisture-free HPLC-grade sodium nitrite standard curve in the range of 0 to 1000 pmol/mL. The difference in absorbance (572–587 nm) was considered for calculations [28].

### 2.12. Tetrahydrobiopterin and Dihydrobiopterin

BH4 and BH2 were determined simultaneously in samples by capillary zone electrophoresis with laser excitation fluorescence detection. For this purpose, the sample was diluted 1:1 with 0.1 M sodium hydroxide, passed through a Sep-Pak Classic NH_2_ cartridge (Waters, Urbana, IL, USA) and analyzed directly using P/ACEMDQ System capillary electrophoresis equipment (Beckman Coulter, Redlands, CA, USA) in which the capillary was preconditioned by passing a 1.0 M sodium hydroxide solution for 30 min, followed by deionized water (Hycel Reactivos Químicos, S.A. de C.V., Zapopan, Jalisco, Mexico) for 30 min, and finally running buffer (0.1 M Tris + 0.1 M boric acid + 2 mM EDTA, pH 8.75) for 30 min. Samples were injected under hydrodynamic pressure at 0.5 psi/10 s. Separation was performed at 20 kV for 10 min with laser excitation at 448 nm and detection at 435 nm. Capillary was washed between runs with 1.0 M NaOH for 2 min and deionized water (Hycel Reactivos Químicos, S.A. de C.V., Zapopan, Jalisco, Mexico) for 2 min. Results are expressed in pmol/mL. BH4 and BH2 concentrations were determined using a standard curve, separately for dihydrobiopterin and tetrahydrobiopterin [29].

### 2.13. Total Antioxidant Capacity

A total of 35 µL of sample was placed in a 96-well plate. Subsequently, 145 µL of 0.1 M phosphate buffer at pH 7.5 was added and homogenized at 500 rpm for 200 s. Immediately after, 100 µL of diluted sample was transferred to an adjacent well, and 50 µL of 0.01 M CuCl_2_ was added and homogenized at 500 rpm for 200 s. Next, 50 µL of 0.01 M bathocuproine was added and homogenized again at 500 rpm for 200 s. Both samples (diluted sample and treated sample) were analyzed spectrophotometrically at a wavelength of 490 nm, after adjusting the equipment with a phosphate buffer blank (UV-1800 spectrophotometer, Shimadzu L.A., S.A., Buenos Aires, Argentina). TAC is expressed in µmol/L and was calculated using a standard curve, for which a normal control serum (8002101 Normal Control Serum N, Valtek, Nuñoa, Metropolitan Region, Chile) was used [30].

### 2.14. Oxidizing Capacity

OxCap determination was performed using a commercial kit. In a 96-well plate, 50 µL of sample and 50 µL of isotonic saline at 37 °C were added, gently homogenized for 1 min at 200 rpm, and 100 µL of freshly prepared Mito-ID MP reagent (Enzo Life Sciences, Farmingdale, NY, USA, Cat.: ENZ-51018-0025) was added. The mixture was gently homogenized for 1 min at 200 rpm and incubated at room temperature for 30 min, protected from light. A total of 100 µL of 50 mM phosphate buffer, pH 7.5, was added and gently homogenized for 1 min at 200 rpm. A total of 20 µL of CCCP (Carbonyl Cyanide *m*-Chlorophenylhydrazone) reagent prepared in 50 mM citrate buffer was added at a concentration of 4 µM. The reagent was homogenized at 200 rpm until orange. Spectrophotometric reading was then carried out at an excitation wavelength of 590 nm and an emission wavelength of 620 nm (Cary Eclipse spectrophotometer, Varian, Pty Ltd., Mulgrave, Victoria, Australia). A control without CCCP reagent was run, with greater cytotoxicity, higher consumption of Mito-ID MP, and a lower intensity of orange color of the reaction. OxCap is expressed in pmol/L.

### 2.15. Interleukin-6

IL-6 was determined in samples using a commercial kit (IL-6 Rat ELISA Kit, Invitrogen, Thermo Fisher Scientific, Cat.: BMS625, Palo Alto, CA, USA).

### 2.16. Alpha Tumor Necrosis Factor

TNF-α was determined in samples using a commercial kit (Rat TNF alpha ELISA Kit, Abcam, Cat.: ab100785, Toronto, ON, Canada).

### 2.17. Malondialdehyde and Malonate

MDA and MTO were determined simultaneously in samples by capillary zone electrophoresis. The sample was diluted 1:2 with cold 0.1 M sodium hydroxide and analyzed directly. For this purpose, a Beckman Coulter P/ACEMDQ system (Urbana, IL, USA) was used, and it was capillary preconditioned by passing a 0.1 M sodium hydroxide solution at 20 psi for 10 min, followed by deionized water for 10 min, and finally, running buffer (10 mM borates + 0.5 mM CTAB at pH 9.0) for 10 min. Samples were injected under hydrodynamic pressure at 0.5 psi/10 s. Separation was performed at −25 KV for 4 min at 267 nm. The capillary was rinsed between runs at 20 psi with 0.1 M NaOH for 2 min, distilled water for 2 min, and running buffer for 4 min. MDA and MTO concentrations are expressed in pmol/mL and were determined separately using respective standard curves [31].

### 2.18. 8-Hidroxy-2-deoxiguanosine

8HO2dG was determined in samples by capillary zone electrophoresis and diode array UV detection. The sample was deproteinized with 20% trichloroacetic acid in a 2:1 ratio. It was centrifuged at 16,000× *g* for 15 min at 4 °C and filtered through a 0.22 µm nitrocellulose membrane filter (Waters, Palo Alto, CA, USA), diluted 1:2 with 2 M sodium hydroxide, and analyzed directly using a P/ACE MDQ system from Beckman Coulter (Urbana, IL, USA), which was preconditioned by passing a 2 M sodium hydroxide solution for 30 min, then deionized water for 30 min, and finally, running buffer (10 mM borates at pH 9.0) for 30 min. Samples were injected under hydrodynamic pressure at 0.5 psi/10 s, and separation was performed at 20 kV for 8 min at 200 nm. The capillary was washed between runs with 2 M sodium hydroxide for 2 min and deionized water for 2 min. Results are expressed in pmol/mL. 8HO2dG concentration was determined using a standard curve [32].

### 2.19. Calcitonin Gene-Related Peptide Quantification

CGRP was determined in tissue samples by high-pressure liquid chromatography (UPLC) with UV-Vis detection using a diode array. Tissue homogenate was first deproteinized with cold methanol (5:1 *v*/*v*) and then with cold 20% trichloroacetic acid (5:1 *v*/*v*). It was centrifuged at 16,000× *g* for 15 min at 4 °C and filtered through 0.22 µm nitrocellulose membrane filters (Waters, Palo Alto, CA, USA), diluted 1:1 (*v*/*v*) with cold 0.01 M sodium hydroxide, passed through a SepPak Classic C-18 cartridge (Waters, Palo Alto, CA, USA), previously treated with 10 mL of 100 mM phosphate buffer at pH 2.5, and analyzed directly with the Waters Acquity UPC H-Class system (Beckman Coulter, Urbana, IL, USA), which had been preconditioned on column (UPLC Acquity HSS 1.8 µm × 5 cm × 2 mm, Waters, Palo Alto, CA, USA) by passing an acetonitrile–water mobile phase (5:95) for 80 min at a flow rate of 0.2 mL/min. A total of 10 µL of sample was injected, and a run was performed at 0.2 mL/min with the same preconditioning mobile phase for 60 min at 40 °C, with UV-Visible detection at 254 nm. CGRP concentration was determined using a standard curve [33].

### 2.20. Protein Expression for TRPV1, eNOS, SOD, and Catalase

Left ventricular tissue was homogenized in lysis buffer (Ripa modified), and a protease/phosphatase inhibitor cocktail (Thermo Scientific, Palo Alto, CA, USA) was added. Homogenate was centrifuged at 14,000 rpm for 15 min at 4 °C. Supernatant was separated and stored at −70 °C until the day of analysis. The Bradford method was used to determine total protein concentration in the homogenate [34]. The equivalent of 50 µg of protein was used for the assay. Acrylamide/bis-acrylamide gels were used for this study. Blots were performed on polyvinylidene difluoride (PVDF) membranes. Transfer was blocked for 1 h at room temperature using TBS-0.01% Tween plus 5% skim milk. Unbound membranes were incubated overnight at 4 °C with primary antibodies rabbit anti-TRPV1 (V2764, Sigma-Aldrich, St. Louis, MO, USA), anti-eNOS (sc-376751, Santa Cruz Biotechnology, Palo Alto, CA, USA), anti-p-eNOS (sc-136519, Santa Cruz Biotechnology, Palo Alto, CA, USA), catalase (sc-271803, Santa Cruz Biotechnology, Palo Alto, CA, USA), SOD-1 (sc-271014, Santa Cruz Biotechnology, Palo Alto, CA, USA), and SOD-2 (sc-137254, Santa Cruz Biotechnology, Palo Alto, CA, USA). All blots were incubated with anti-β-actin antibody (sc-81178, Santa Cruz Biotechnology, Palo Alto, CA, USA) as a loading control. Film images were obtained digitally using a GS-800 densitometer and Quantity One Software (Version 4.6.8) (Both Bio-Rad Laboratories, Inc., Hercules, CA, USA). Reports are in arbitrary units (AU).

### 2.21. Histological Analysis

After sacrifice, hearts were removed from all experimental groups, washed with 0.9% saline, fixed with 10% formalin/PBS (Santa Cruz Biotechnology, Palo Alto, CA, USA), and embedded in paraffin (Santa Cruz Biotechnology, Palo Alto, CA, USA) to obtain 2 μm cross sections. These sections were mounted on glass slides for immunolocalization of TRPV1 and CGRP using IHC technique. Glass slides were deparaffinized (98% xylene) and rehydrated (alcohol gradient [100%, 95%], with distilled water and 1X PBS pH 7.2 (NaCl 1 g, KCl 0.25 g, Na_2_HPO_4_ 1.42 g, and KH_2_PO_4_ 0.25 g). They were then placed in a water bath for 5 min in a sodium citrate solution (2.94 g in 1X PBS) and allowed to cool to room temperature for 10 min. Endogenous peroxidase activity was blocked with 3% hydrogen peroxide in methanol and then with 1.5% serum albumin (Sigma Aldrich, St Louis, MO, USA) for 2 h. Slides were incubated with their respective primary antibody (TRPV1, diluted 1:100 in 1.5% albumin buffer, CGRP, diluted 1:100 in 1.5% albumin buffer, in a humid chamber under refrigeration until the following day. Three washes were performed with PBS 1X pH 7.2 solution for 10 min each and secondary antibody (Anti Rabbit, diluted 1:100 in 1.5% albumin buffer). It was incubated for 2 h in a humid chamber at room temperature. Antigen–antibody complex was visualized using a 3,3’-diaminobenzidine 8 kit (Dako, Agilent Technologies, Glostrup, Denmark). Slides were then mounted using Permount mounting medium (17986-01 Electron Microscopy Sciences, Urbana, IL, USA) and coverslips (24 × 40 mm, Corning, Palo Alto, CA, USA). Negative controls were obtained using the same experimental procedure, and only the corresponding primary antibody was omitted, so the secondary antibody did not show any labeling.

Panoramic images were captured at 10X magnification to locate areas where the brown DAB stain of each condition was located, using an Olympus BX51 microscope (Center Valley, PA, USA). Images were captured using OLYMPUS CellSens 1.18 software (Center Valley, PA, USA).

In a double-blind design and in order to confirm distribution of positivity of antigen–antibody complex, a full scan was performed at 40X magnification of histological sections resulting from immunostaining for TRPV1 and CGRP, capturing images with a camera (Q IMAGING, Micro Publisher 5.0; REUZEit, Temecula, CA, USA), interfaced with Pro Premier version 9 software (Media Cybernetics; Rockville, MD, USA), coupled to an Olympus BX51 microscope (Olympus; Hachioji, Tokio, Japón ). Slices were scanned from each animal, from each condition [one slice per animal (n = 3), 2 slices/animal and 4 images/slice/4 regions (external, internal, septal myocardium, and endocardium).

### 2.22. Statistical Analysis

Results are expressed as mean ± standard error of mean (SEM) of 3–6 independent experiments. For multiple comparisons, we applied one-way or two-way analysis of variance (ANOVA), followed by Tukey’s post hoc test (Sigma Plot 13 software, Jandel Scientific, San Jose, CA, USA). Statistical significance was set at *p* < 0.001 [35].

## 3. Results

### 3.1. Absorption of Capsaicin

**Figure 2:**
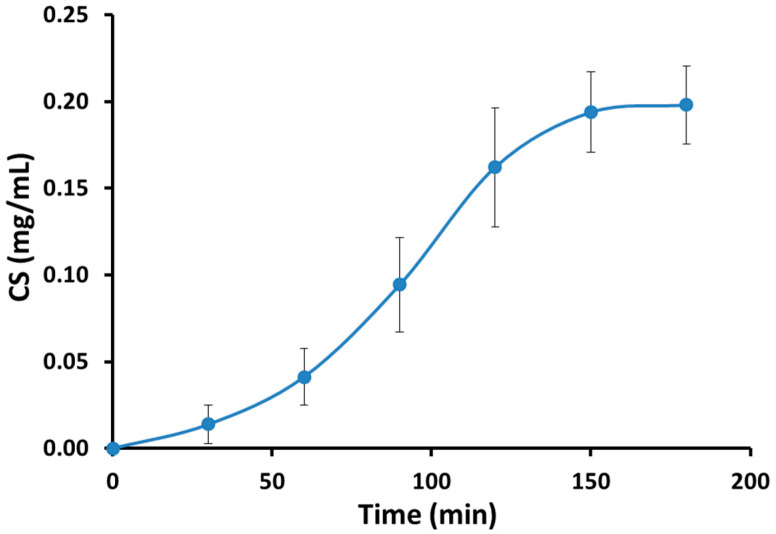
Plasmatic levels of capsaicin. The curve shows the peak plasma level of CS at 3 h (180 min), after topical administration of 2 g of cream containing 0.6 mg of CS. Final plasma concentration of CS at 3 h was 0.1983 ± 0.0223 mg/mL (33.05 ± 3.71% of initial dose). Data are expressed as mean ± SE; n = 4.

Figure 2 shows the time course of plasma CS over a 3 h period (180 min) after topical administration (2 g cream, 0.6 mg CS). Final plasma CS concentration at 3 h was 0.1983 ± 0.0223 mg/mL, equivalent to 33.05 ± 3.71% of the initial dose.

### 3.2. Mean Arterial Pressure

**Figure 3:**
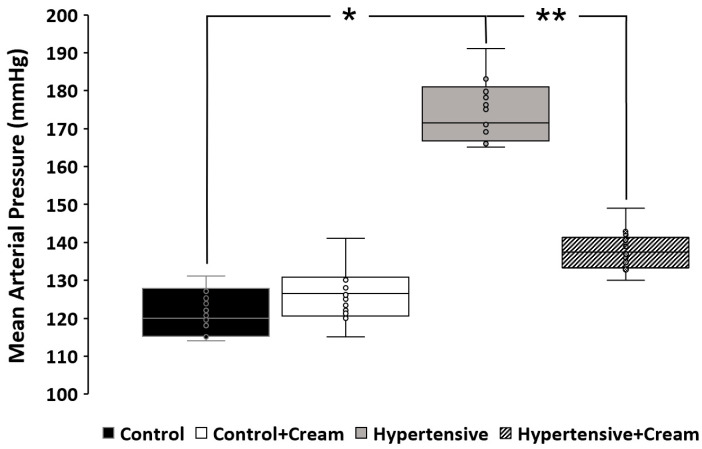
Mean arterial pressure (mmHg). Mean arterial pressure of animals from different experimental groups is shown. Data are expressed as mean ± SE; * *p* < 0.001, statistically significant compared to Control vs. Hypertensive; ** *p* < 0.001, statistically significant compared to Hypertensive vs. Hypertensive+Cream. One-way ANOVA followed by a Tukey’s post hoc test; n = 8.

Mean arterial pressure (MAP) (Figure 3) of different groups of animals was as follows: (1) Control = 122.83 ± 2.89 mmHg, (2) Control+Cream = 128.33 ± 3.46 mmHg, (3) Hypertensive = 173 ± 4.47 mmHg, and (4) Hypertensive+Cream = 136.83 ± 2.72 mmHg. Increase in mean arterial pressure at the end of treatment in the Control+Cream group was probably due to the direct impact of capsaicin on healthy vascular smooth muscle, causing constriction due to increased calcium influx, which may be the cause that led to an elevation in MAP in Control+Cream rats [25].

### 3.3. Cardiac Mechanical Work

**Figure 4:**
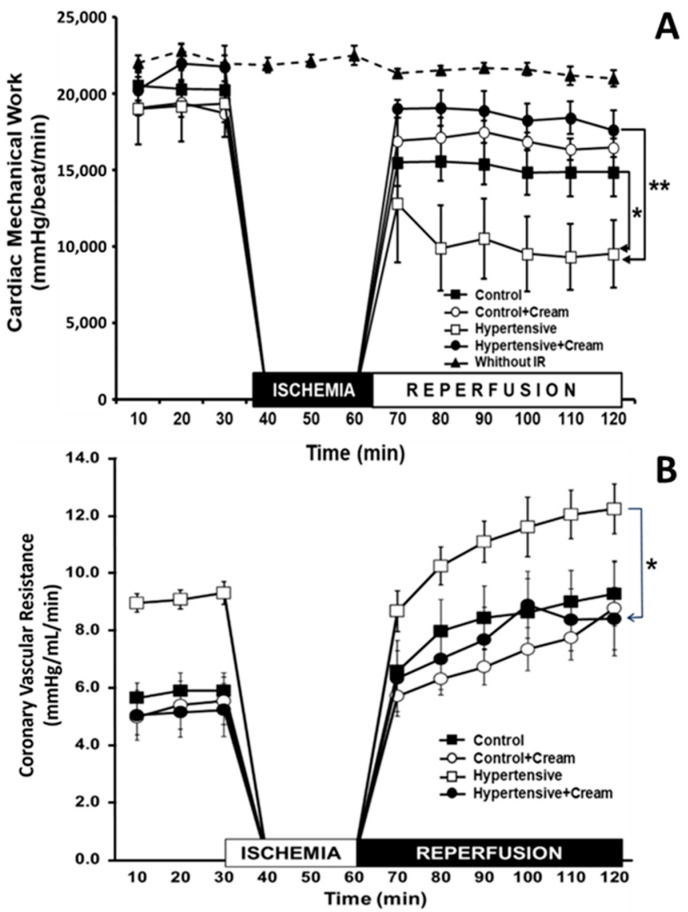
(**A**) Cardiac mechanical work. Study of the performance of isolated and perfused hearts without ischemia-reperfusion (I-R) and exposed to I-R, from rats with and without treatments. This figure shows cardiac work during the pre-ischemic period and differences in cardiac work in control or hypertensive rats and in the absence and presence of CS treatment. Data are expressed as mean ± SE; * *p* < 0.001, statistically significant compared to Control vs. Hypertensive; ** *p* < 0.001, statistically significant compared to Hypertensive vs. Hypertensive+Cream. Two-way ANOVA (Two Factor Repetition) followed by a Tukey’s post hoc test; n = 6. (**B**) Coronary vascular resistance (CVR). This is an index of coronary artery relaxation or contraction. We can observe that treatment with cream improves CVR in the Hypertensive group (Hypertensive+Cream) after I-R. Data are expressed as mean ± SE; * *p* < 0.001, statistically significant compared to Hypertensive vs. Hypertensive+Cream. Two-way ANOVA (Two Factor Repetition) followed by a Tukey’s post hoc test; n = 6.

Figure 4A shows the results obtained from CMW in isolated rat hearts with different treatments. Hearts without I-R maintained the same level of CMW throughout the two hours of the experiment. Compared with this group, the CMW of the Control group hearts exposed to I-R was significantly decreased by 29% on average during reperfusion. This same behavior was observed in the Control+Cream group hearts, with a 21% decrease in CMW. In turn, in the hearts of hypertensive rats, CMW decreased by 52%, and in the treated Hypertensive group (Hypertensive+Cream), it decreased by 13% during reperfusion. When comparing the Hypertensive vs. Hypertensive+Cream groups, a significant increase in CMW was observed after treatment with CS.

On the other hand, CVR is an index of relaxation or contraction of the coronary arteries. In Figure 4B, during the pre-ischemic period, it can be observed that, in the Hypertensive group, CVR increased significantly compared to Control, Control+Cream, and Hypertensive+Cream groups. This same trend was observed after ischemia, when reperfusion occurred. However, treatment with cream in the Hypertensive group (Hypertensive+Cream) prevented CVR from increasing. Thus, we see that the mechanical work of the heart is directly related to CVR; that is, better mechanical work results in better vascular resistance.

### 3.4. Effects of CS on Oxidation of BH4 to BH2 and on Production of NO, TAC, and OxCap

Table 1 and Table 2 show results obtained at both systemic and tissue levels for the BH4/BH2 ratio, which demonstrates oxidation of BH4 to BH2 during experimental hypertension. It is also observed how CS prevents this oxidation. Furthermore, treatment with CS also favored a reduction in BH2 to BH4 (Hypertensives vs. Hypertensives + cream). Respective concentrations of both biopterins are also shown.

On the other hand, a decrease in BH4 levels in the Hypertensive group was reflected in NO production (Table 1). This was due to a decrease in the viability of total antioxidant capacity at the endogenous level (TAC, from 285.30 ± 32.67 to 93.46 ± 22.07 mmol/L), which resulted in an increase in systemic oxidation (OxCap) (from 0.19 ± 0.035 to 0.47 ± 0.076 pmol/L) (Table 1). The same behavior was observed at the tissue level (Table 2).

Treatment with CS in rats in the Hypertensive group (Hypertensive+Cream) significantly improved TAC (from 93.46 ± 22.07 to 171.48 ± 30.40 mmol/L), resulting in a decrease in systemic OxCap (from 0.47 ± 0.076 to 0.33 ± 0.053 pmol/L) with a consequent increase in NO (from 9.99 ± 0.76 to 21.67 ± 0.88 mmol/L), compared to the Hypertensive group at a systemic level (Table 1). Same results were observed in myocardial tissue (Table 2).

### 3.5. Effect of CS at a Systemic Level on Cell Damage Molecules (MDA and MTO) and DNA Damage (8HO2dG)

Table 1 shows that MDA, MTO, and 8HO2dG levels are increased in *L*-NAME-induced hypertension compared with the Control group. This alteration is also significantly reversed after treatment with CS.

### 3.6. Effect of CS on Inflammatory Biomarkers Such as Tumor Necrosis Factor (TNF-α) and Interleukin-6 (IL-6) in Ventricular Tissue

TNF-α and IL-6 at a systemic level were significantly increased in the Hypertensive group from 28.33 ± 5.16 to 116.94 ± 7.53 pg/mL and from 106.01 ± 10.88 to 159.91 ± 1.01pg/mL, respectively, compared to the Control group (Table 1). After CS treatment, TNF-α and IL-6 levels decreased significantly (from 116.94 ± 5.16 to 34.24 ± 8.05 pg/mL and from 159.91 ± 10.39 to 131.62 ± 7.50 pg/mL, respectively) (Table 1).

Something similar was observed at a tissue level (Table 2), where TNF-α and IL-6 increased significantly in the Hypertensive group compared to the Control group (TNF-α 10.70 ± 0.83 to 122.97 ± 27.40 pg/mL; IL-6 3.73 ± 0.87 to 232.81 ± 1.02 pg/mL) and, after treatment with CS (Hypertensive+Cream), levels of these biomarkers decreased significantly (TNF-α 122.97 ± 22 to 3.21 ± 3.60 pg/mL; IL-6 22.81 ± 1.02 to 7.02 ± 1.31 pg/mL) (Table 2).

### 3.7. Effect of CS in Left Ventricular Tissue of Hypertensive Rats

Table 2 shows results obtained from the evaluation of levels of biomarkers NO, BH4, BH2, TAC, OxCap, 8HO2dG, TNFα, and IL-6 in cardiac tissue. Likewise, the observed BH4/BH2 ratio indicates a degree of oxidation of BH4 to BH2 during experimental hypertension (Hypertensive) compared to the Control group (Control). Treatment with CS significantly reduced oxidation of BH4 to BH2 during hypertension (Hypertensive+Cream).

On the other hand, CS improved BH4 levels by reducing BH2 formation in the Hypertensive+Cream group compared to the Hypertensive group (Table 2). It was also observed that *L*-NAME-induced HAS decreased NO levels (from 38.79 ± 6.23 to 4.93 ± 1.06 pmol/mL) and TAC (decreased from 501.62 ± 33.26 to 158.42 ± 22.52 mmol/L), consequently with *L*-NAME, and an increase in OxCap was observed (from 0.10 ± 0.0056 to 0.30 ± 0.058 pmol/L), compared to the Control group (Table 2).

After treatment with CS (Table 2), both NO and TAC increased significantly (from 4.93 ± 1.06 to 17.06 ± 2.37 mmol/L and from 158.42 ± 22.52 to 585.61 ± 48.74 mmol/L, respectively), while OxCap decreased significantly in the Hypertensive+Cream group compared to the Hypertensive group (Table 2).

### 3.8. Effect of CS on Cell Damage Molecules Such as MDA, MTO, and DNA Damage, Such as 8HO2dG in Ventricular Tissue

Table 2 shows changes observed in MDA, MTO, and 8HO2dG levels due to SAH generated in animals in the Hypertensive group. A significant increase in MDA and MTO, as well as 8HO2dG, was evident compared to the Control group. CS administration resulted in a significant decrease in evaluated biomarkers (MDA, MTO, and 8HO2dG) (Hypertensive+Cream), compared to the Hypertensive group (Table 2).

### 3.9. Effect of CS on CGRP Levels in Ventricular Tissue

**Figure 5:**
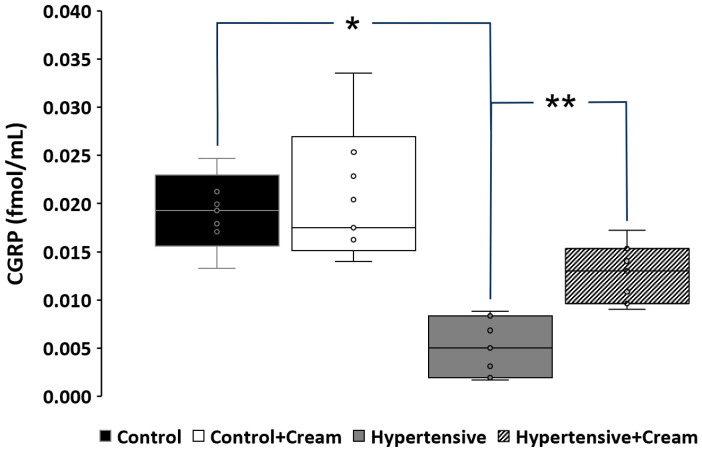
CGRP levels in ventricular tissue in rats from different groups: control (black bar), control with CS treatment (white bar), hypertensive (grey bar), and hypertensive plus capsaicin treatment. Data are expressed as mean ± SE; * *p* < 0.001, statistically significant compared to Control vs. Hypertensive; ** *p* < 0.001, statistically significant compared to Hypertensive vs. Hypertensive+Cream. One-way ANOVA followed by a Tukey’s post hoc test; n = 5.

Figure 5 shows a significant decrease in CGRP in the Hypertensive group (from 0.019 ± 0.0019 to 0.0051 ± 0.0015 fmol/mL), compared to the Control group. CS increased CGRP levels in cardiac tissue (from 0.0051 ± 0.0015 to 0.0128 ± 0.0016 fmol/mL), in animals in the treated Hypertensive group (Hypertensive+Cream).

### 3.10. Expression of Molecules Related to Control of Oxidative Stress

**Figure 6:**
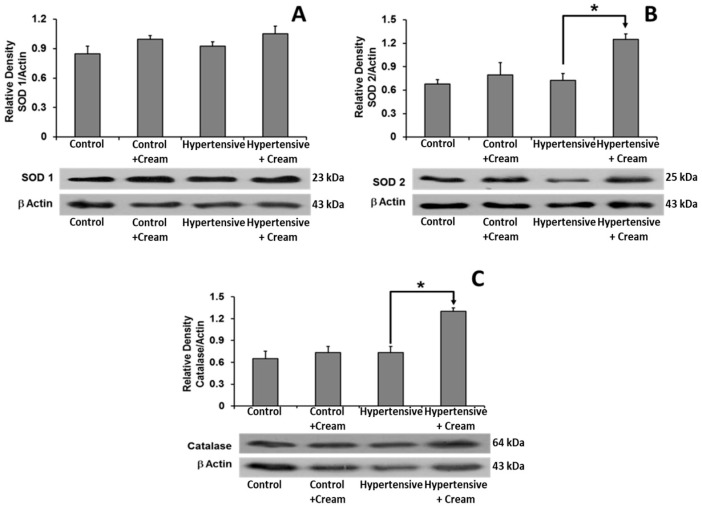
Effect of capsaicin cream treatment on expression of antioxidant enzymes in ventricular tissue. Representative Western blots and quantification of protein expression levels of superoxide dismutase 1 (SOD1) (**A**), superoxide dismutase 2 (SOD2) (**B**), and catalase (**C**) in ventricular tissue of four groups: Control, Control+Cream, Hypertensive, and Hypertensive treated with capsaicin cream (Hypertensive+Cream). Data are expressed as mean ± SE; * *p* < 0.001, statistically significant compared to Hypertensive vs. Hypertensive+Cream. One-way ANOVA followed by a Tukey’s post hoc test; n = 3.

Superoxide dismutase (SOD1) showed no significant differences between treated groups. However, SOD2 and catalase levels showed significant increases in the Hypertensive+Cream group compared to the Hypertensive group (Figure 6).

**Figure 7:**
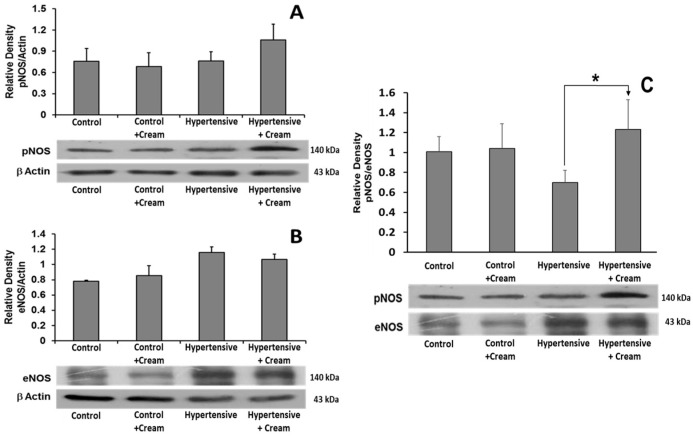
Effect of capsaicin cream treatment on expression of phosphorylated nitric oxide synthase (pNOS) and endothelial nitric oxide synthase (eNOS) in ventricular tissue. Representative Western blots and quantification of protein expression levels of phosphorylated NOS (pNOS) (**A**), endothelial NOS (eNOS) (**B**), and pNOS/eNOS ratio (**C**) in ventricular tissue from four groups: Control, Control+Cream, Hypertensive, and Hypertensive+Cream. Data are expressed as mean ± SE; * *p* < 0.001, statistically significant compared to Control vs. Hypertensive. One-way ANOVA followed by a Tukey’s post hoc test; n = 3.

Regarding the expression of eNOS and phosphorylated NOS (pNOS), no significant differences were observed between treated groups (Figure 7).

### 3.11. TRPV1 Expression in Ventricular Tissue

**Figure 8:**
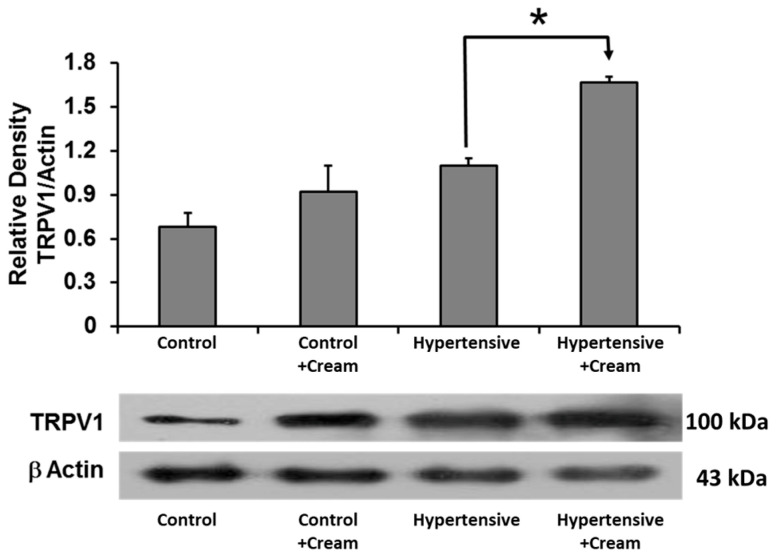
Effect of capsaicin cream treatment on TRPV1 expression in ventricular tissue. Representative Western blot and quantification of TRPV1 protein expression in ventricular tissue from the following groups: Control, Cream, Hypertensive, and Hypertensive treated with capsaicin cream (Hypertensive+Cream). Data are expressed as mean ± SE; * *p* < 0.001, statistically significant compared to Control vs. Hypertensive. One-way ANOVA followed by a Tukey’s post hoc test; n = 3.

TRPV1 expression showed a tendency to increase in the Hypertensive group compared to the Control group, which was not significant. However, TRPV1 increased significantly after CS administration in the Hypertensive+Cream group compared to the Hypertensive group (Figure 8).

### 3.12. Immunolocalization of TRPV1 in Cardiac Tissue

**Figure 9:**
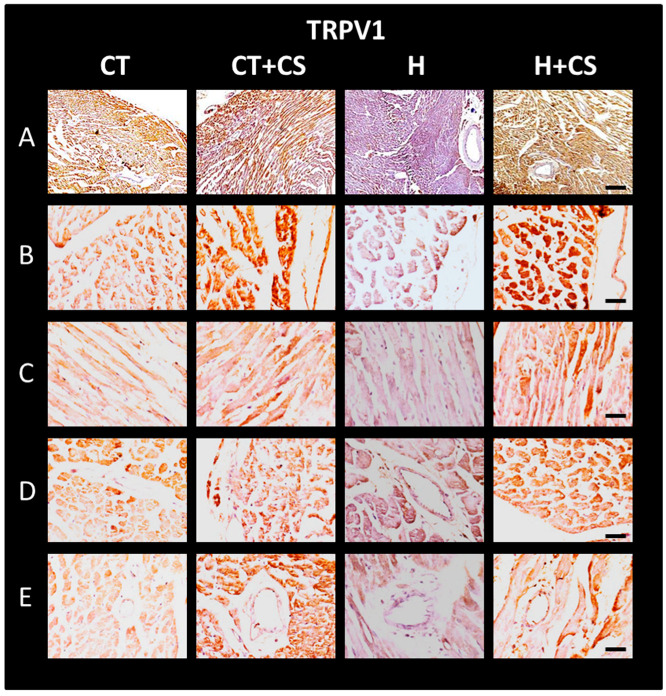
Representative optical micrograph of TRPV1 immunolabeling. Images in (**A**–**E**) were obtained at 10X and 40X, respectively. Compared with the Control group (CT), consistent increased labeling was observed, especially in hypertensive animals treated with capsaicin cream (H+CS), throughout the ventricular wall, including outer (**B**), inner (**C**), septal myocardium (**D**), and endocardium (**E**). Bar in (**A**) = 50 µm and in (**B**–**E**) = 25 µm.

Changes in TRPV1 expression were confirmed by immunolocalization in cardiac tissue (Figure 9). TRPV1 labeling was localized throughout the cardiac wall in both panoramic images (10X) (A) and close-up images (40X) at the external (B), internal (C), septal myocardium (D), and endocardium (E) levels. Results are consistent with those observed with WB (Figure 8).

### 3.13. Immunolocalization of CGPR in Cardiac Tissue

**Figure 10:**
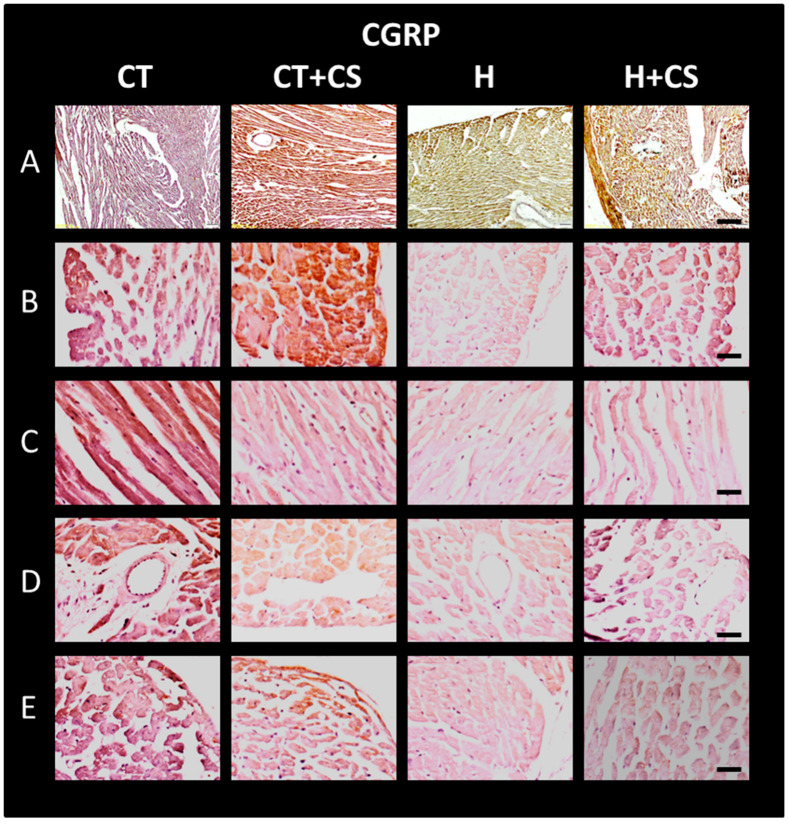
Representative optical micrograph of CGRP immunolabeling. Images in (**A**–**E**) were obtained at 10X and 40X, respectively. Treatment with capsaicin cream (CS) in hypertensive rats (H+CS) increased CGRP expression, compared with the Hypertensive group (H), throughout the ventricular wall, including outer (**B**), inner (**C**), septal myocardium (**D**), and endocardium (**E**). Bar in (**A**) = 50 µm and in (**B**–**E**) = 25 µm.

Changes in CGPR expression were confirmed by immunolocalization in histological sections of cardiac tissue (Figure 10). CGPR labeling was localized throughout the cardiac wall in both panoramic images (10X) (A) and close-up images (40X) at the external myocardium (B), internal myocardium (C), endocardium (D), and septum (E) levels. Compared to control (CT), CGRP immunodetection was lower in group H and higher in CS-treated groups (C+CS and H+CS), as observed by WB (Figure 5).

## 4. Discussion

Systemic arterial hypertension (SAH) disrupts vascular homeostasis primarily through oxidative stress (OS), which leads to endothelial dysfunction, reduced nitric oxide (NO) bioavailability, and tissue injury. One of the key mechanisms implicated in this process is oxidation of tetrahydrobiopterin (BH4) to dihydrobiopterin (BH2), resulting in endothelial nitric oxide synthase (eNOS) uncoupling and impaired NO synthesis. Our results demonstrate that topical administration of capsaicin (CS), a known TRPV1 agonist, effectively mitigates these pathological processes in a rat model of *L*-NAME-induced hypertension (Table 1 and Table 2).

TRPV1 activation by CS appears to restore vascular function through multiple interconnected pathways. Notably, TRPV1 upregulation was observed in cardiac tissue following CS treatment (Figure 8 and Figure 9), suggesting that capsaicin not only preserves TRPV1 expression but also enhances its functional capacity. This aligns with previous studies indicating that TRPV1 modulates intracellular calcium influx and contributes to NO-mediated vasodilation and cardioprotection. In our model, CS treatment preserved BH4 levels, decreased BH2 concentrations, and significantly decreased the BH2/BH4 ratio, both systemically and in ventricular tissue (Table 1 and Table 2), indicating improved eNOS coupling and enhanced NO bioavailability [36].

In hypertensive rats treated with CS, we observed a significant decrease in mean arterial pressure (Figure 3), as well as significant increases in NO, catalase, and superoxide dismutase 2 levels (Table 1 and Table 2, Figure 6), alongside a reduction in systemic and myocardial oxidative capacity (OxCap). These changes were accompanied by a decrease in markers of lipid peroxidation (MDA and MTO) and DNA oxidation (8OH2dG) (Table 1 and Table 2), confirming that CS effectively reduced oxidative damage. The cardioprotective effect of CS was further evidenced by a significant attenuation of ischemia-reperfusion (I/R)-induced cardiac dysfunction, as assessed by the Langendorff model (Figure 4) [11,13,14,16,25].

Our findings also show that TRPV1 activation by CS leads to increased expression and tissue levels of calcitonin gene-related peptide (CGRP) (Figure 5 and Figure 10), a neuropeptide with potent vasodilatory and anti-inflammatory properties. Upregulation of CGRP may represent a key mediator in observed cardioprotection, especially given its role in attenuating inflammatory responses and enhancing endothelial function [1,11,25,37].

Importantly, CS treatment decreased levels of pro-inflammatory cytokines TNF-α and IL-6 in hypertensive rats. These effects were observed both systemically and at the cardiac tissue level (Table 1 and Table 2). Interestingly, the Control+Cream group exhibited a modest increase in inflammatory biomarkers, likely due to transient calcium influx and potential pro-inflammatory signaling induced by TRPV1 activation in non-pathological conditions. This suggests that effects of CS may vary depending on the physiological state of vasculature, with a greater benefit observed in a setting of pre-existing OS and endothelial dysfunction [11,17,18,25,38,39].

The observed increase in TRPV1 and CGRP immunoreactivity in hypertensive rats treated with CS (Figure 9 and Figure 10, panels H-A to H-E) further supports a mechanistic link between TRPV1 signaling and cardioprotection. These effects were predominantly localized to external and internal myocardium, endocardium, and septum, suggesting that TRPV1-mediated modulation of local vascular tone and inflammation may underlie preserved cardiac function following I/R [16,30,40,41,42].

In addition to biological outcomes, the mode of CS delivery is relevant. Topical administration provided sustained systemic effects without the skin irritation commonly observed with subcutaneous injections. Although mild dryness was noted at the application site, no signs of ulceration or scarring were present, supporting its feasibility as a non-invasive therapeutic approach.

**Figure 11:**
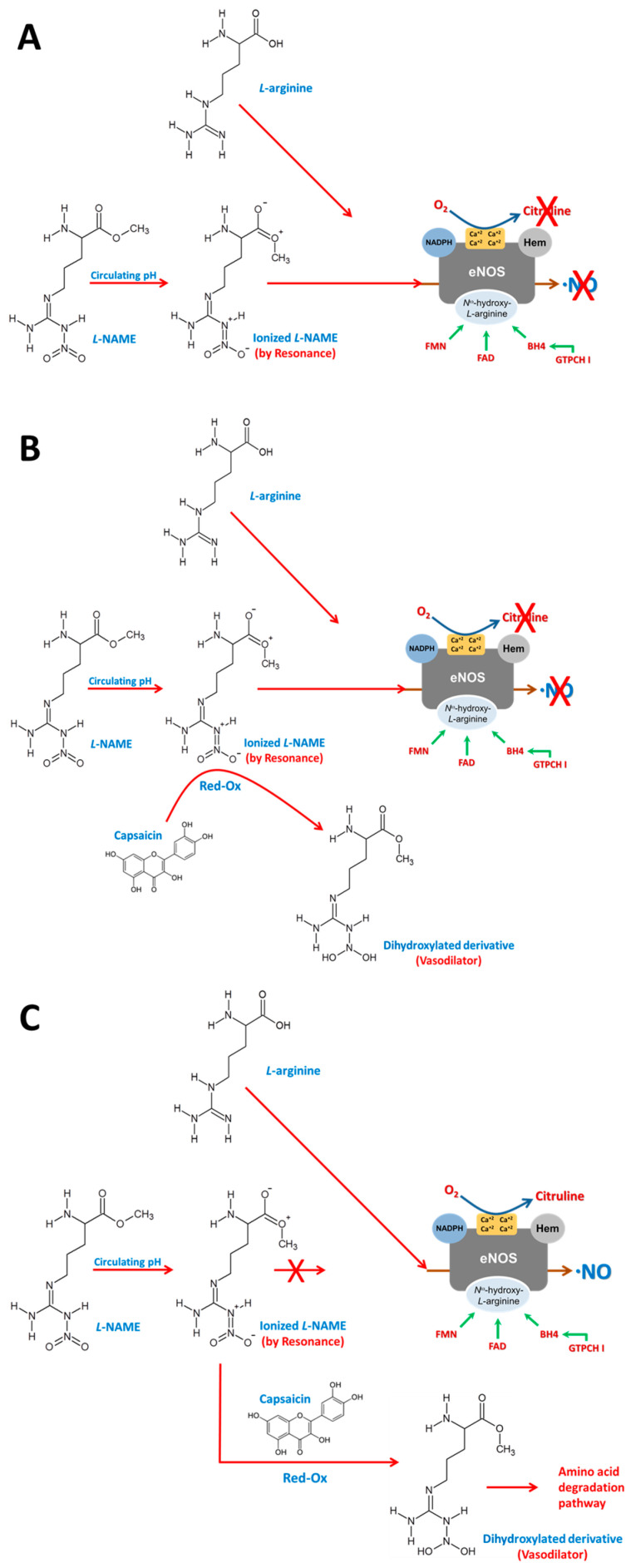
Effect of capsaicin on *L*-NAME. (**A**) When *L*-NAME enters the body, it undergoes ionization due to circulating pH and resonance, allowing it to competitively inhibit *L*-arginine, thus competing for the receptor binding site and thereby inhibiting NO synthesis. (**B**) CS, being a polyphenol with an oxidoreductive effect, reduces the circulating ionized *L*-NAME molecule, transforming it into a dihydroxylated derivative with a vasodilatory effect and no affinity for a receptor. (**C**) When CS reduces the circulating ionized *L*-NAME molecule, *L*-arginine is no longer inhibited and, therefore, binds to the enzyme receptor, restoring NO synthesis.

Finally, we propose a chemical hypothesis for the observed restoration of NO synthesis in the presence of *L*-NAME. Based on the redox properties of CS, we suggest that CS may chemically reduce ionized *L*-NAME to a less active derivative, thereby alleviating its competitive inhibition of eNOS and allowing L-arginine binding to resume (Figure 11). This mechanism, while hypothetical, is consistent with improved NO bioavailability and reduced OS markers observed in our study.

Overall, these findings underscore the therapeutic potential of low-dose topical CS as a non-pharmacologic intervention in SAH. By targeting TRPV1-mediated pathways, CS not only restores endothelial function but also enhances myocardial resilience to I/R injury. Future studies should investigate the translational potential of this strategy in clinical models of hypertension and ischemic heart disease.

## 5. Conclusions

Based on the research conducted, we can conclude that the topical application of CS modulates altered mechanisms during OS events, particularly those generated by SAH. This is possible because CS regulates the endogenous antioxidant system by reducing circulating OS, both at the extracellular and intracellular levels. This effect allows TRPV1 to regulate intermembrane Ca^2+^ transport, by reducing ROS that alter such transport in receptors. This allows for regulation of NO production and activation of various NO-dependent vasorelaxation pathways, including the NO/GC pathway. Both the regulation of NO production by CS and that of Ca^2+^ transport through TVPR1 receptors stimulate the expression of various peptides important for cellular and systemic bioavailability and integrity, which regulate levels of inflammatory molecules and damage to tissues and DNA. Among them is CGRP, which mediates vasodilation and cardioprotection, mainly by acting on CGRP receptors.

## 6. Limitations of This Study

One of the main limitations of this study is the use of an animal model, specifically hypertensive rats. Although it allows for rigorous control of physiological variables and offers preliminary evidence of the efficacy of topical capsaicin, it restricts the direct extrapolation of the results to a human clinical setting. There are significant differences between species in cardiovascular regulation, cutaneous response to capsaicin, and the mechanisms involved in oxidative stress, a key component in the pathophysiology of hypertension that could also influence treatment response. Furthermore, although relevant changes in markers of oxidative stress, cellular damage, and inflammation were observed, the duration of this study may not be sufficient to evaluate potential compensatory mechanisms within the body. Therefore, a study with a longer follow-up will be necessary to further explore the relationship between topical capsaicin, blood pressure regulation, oxidative stress, cellular damage, and inflammation.

## Figures and Tables

**Table 1:** Systemic level.

	Control	Control+Cream	Hypertensive	Hypertensive+Cream
	Biomarkers of Nitric Oxide Bioavailability			
**NO** (pmol/mL)	28.05 ± 2.72	18.90 ± 2.44 *	9.99 ± 0.76 **	21.67 ± 0.88 ***
**BH4** (pmol/mL)	6.43 ± 0.51	5.02 ± 0.14 *	3.46 ± 0.11 **	5.64 ± 0.34 ***
**BH2** (pmol/mL)	4.30 ± 0.14	6.33 ± 0.64 *	7.87 ± 0.60 **	5.11 ± 0.20 ***
**BH4/BH2**	1.49 ± 0.09	0.78 ± 0.06 *	0.44 ± 0.07 **	1.10 ± 0.19 ***
**BH2/BH4**	0.67 ± 0.06	1.28 ± 0.15 *	2.27 ± 0.13 **	0.91 ± 0.28 ***
**TAC** (mmol/mL)	285.30 ± 32.67	233.79 ± 8.86 *	93.45 ± 22.07 **	171.48 ± 30.40 ***
**OxCap** (pmol)	0.191 ± 0.035	0.201 ± 0.012	0.471 ± 0.076 **	0.339 ± 0.053 ***
	**Damage Molecules**			
**MDA** (pmol/mL)	0.163 ± 0.011	0.172 ± 0.010	0.69 ± 0.09 **	0.42 ± 0.01 ***
**MTO** (pmol/mL)	0.062 ± 0.005	0.056 ± 0.006	0.233 ± 0.031 **	0.173 ± 0.021 ***
**8HO2dG** (pM)	1.914 ± 0.141	0.99 ± 0.23 *	4.82 ± 0.32 **	3.16 ± 0.29 ***
	**Inflammatory Molecules**			
**TNF-α** (pg/mL)	28.33 ± 5.16	54.43 ± 5.85 *	116.94 ± 7.53 **	34.24 ± 8.05 ***
**IL-6** (pg/mL)	106.01 ± 10.88	100.84 ± 8.02	169.91 ± 10.38 **	131.61 ± 7.50 ***

Systemic levels of biomarkers of nitric oxide bioavailability, damage molecules, and inflammatory molecules in control and hypertensive rats in the presence and absence of capsaicin treatment. Data are expressed as mean ± SE; * *p* < 0.001, statistically significant compared to Control vs. Control+Cream; ** *p* < 0.001, statistically significant compared to Control vs. Hypertensive; *** *p* < 0.001, statistically significant compared to Hypertensive vs. Hypertensive+Cream. One-way ANOVA followed by a Tukey’s post hoc test; n = 5.

**Table 2:** Ventricular tissue.

	Control	Control+Cream	Hypertensive	Hypertensive+Cream
	Biomarkers of Nitric Oxide Bioavailability			
**NO** (pmol/mL)	38.79 ± 6.23	36.36 ± 5.52	4.93 ± 1.06 **	17.06 ± 2.39 ***
**BH4** (pmol/mL)	3.88 ± 0.18	3.74 ± 0.09	2.80 ± 0.02 **	3.17 ± 0.05 ***
**BH2** (pmol/mL)	3.44 ± 0.23	3.39 ± 0.82	11.53 ± 1.30 **	4.60 ± 0.37 ***
**BH4/BH2**	1.13 ± 0.22	0.85 ± 0.36	0.24 ± 0.21 **	0.69 ± 0.29 ***
**BH2/BH4**	0.89 ± 0.31	1.18 ± 0.44	4.11 ± 1.02 **	1.45 ± 1.26 ***
**TAC** (mmol/mL)	501.62 ± 33.26	512.49 ± 37.36	158.42 ± 22.52 **	586.61 ± 48.74 ***
**OxCap** (pmol)	0.1001 ± 0.0056	0.092 ± 0.066	0.302 ± 0.058 **	0.140 ± 0.031 ***
	**Damage Molecules**			
**MDA** (pmol/mL)	0.0241 ± 0.0008	0.0232 ± 0.0012	0.0681 ± 0.0121 **	0.0382 ± 0.0020 ***
**MTO** (pmol/mL)	0.00115639 ± 0.00003008	0.01128842 ± 0.00001449	0.00179634 ± 0.00013201 **	0.00120057 ± 0.00001192 ***
**8HO2dG** (pM)	0.00110238 ± 0.00003053	0.00109327 ± 0.00001243	0.01713044 ± 0.00001322 **	0.00127837 ± 0.00001981 ***
	**Inflammatory Molecules**			
**TNF-α** (pg/mL)	10.70 ± 0.83	27.05 ± 7.33 *	122.97 ± 27.40 **	38.21 ± 3.60 ***
**IL-6** (pg/mL)	3.73 ± 0.87	13.96 ± 2.11 *	22.81 ± 1.02 **	7.02 ± 1.31 ***

Ventricular tissue levels of biomarkers of nitric oxide bioavailability, damage molecules, and inflammatory molecules in control and hypertensive rats in the presence and absence of capsaicin treatment. Data are expressed as mean ± SE; * *p* < 0.001, statistically significant compared to Control vs. Control+Cream; ** *p* < 0.001, statistically significant compared to Control vs. Hypertensive; *** *p* < 0.001, statistically significant compared to Hypertensive vs. Hypertensive+Cream. One-way ANOVA followed by a Tukey’s post hoc test; n = 5.

## Data Availability

All data generated or analyzed during this study are included in this published article and its additional information files.
